# Becquerel’s Galvanic Apparatus

**Published:** 1862-07

**Authors:** 


BECQUEREL'S GALVANIC APPARATUS.—Dr. GARRATT exhibited a new, portable galvanic apparatus, invented and sent to him by Becquerel. It was contained within a box about ten inches by six, and two inches deep. The action was generated in two small cup-cells, the outer carbon, the inner zinc, by a strong solution of bisulphate of mercury prepared instantly by adding to a little of the dry salt a teaspoonful of water in each cell. Attached to it was a helix of very fine silver wire, by the adjustment of which the power was regulated. It was so arranged as to produce both a modified primary and induced current. Its full effect was very powerful. Dr. Garratt had applied to the original metallic handles variously shaped and ingeniously contrived electrodes, coated with caoutchouc and tipped with sponge, so as to prevent accidental shocks both to exhibiter and patient. By their employment currents could be applied to all parts of the body without the necessity of removing the garments, and thus one of the chief objections to the use of galvanism is removed. In amenorrhœa Dr. Garratt applied it externally only, and sometimes with marked success; in other cases, apparently quite as appropriate for its use, without any benefit at all.

In reply to a question from Dr. HOMANS, he stated that he had found galvanism to be most useful in certain cases of rheumatism, those particularly in which the application of cold was grateful, and in cases of nervous exhaustion, however produced. In the extraction of teeth it seemed available, if properly applied. The current should be applied at the proper instant, and the instrument must fit the tooth exactly. The relief from pain seems to be caused by pre-occupation of the nerve at the moment the tooth is pulled; for if the operation is at all protracted, the effect ceases.

Dr. ABBOT mentioned a case in which he had recently employed magnet electricity against narcotism produced by laudanum. The patient, a healthy woman of middle age, had swallowed three drachms of laudanum at 7½ o'clock in the morning, for the purpose of self-destruction. Two hours afterwards, a physician in the neighborhood was called, who gave an emetic, but the matter vomited at that time and subsequently gave no smell of opium. Dr. Abbot first saw the patient at 10½ o'clock, A. M. At this time there was complete loss of consciousness, with contracted pupils, respiration fifteen and pulse seventy in a minute. Strong coffee was poured down her throat until a certain amount had accumulatek in the stomach, when it was rejected by vomiting. It was impossible to walk her about, as she had no power in her limbs whatever. The occasional vomiting served to stimulate the heart's action from time to time until 4, P. M., when the pulse had become very feeble, the respiration only seven in a minute, the face haggard, the jaw fallen, and deglutition no longer possible. The poles of the battery were then placed in her hands, and the shock at once excited a long, sonorous inspiration, the chest expanding to its utmost. A succession of rapid and full inspirations followed as long as the battery was applied, terminating, if the application was continued with considerable force, in a spasmodic closure, with rapid convulsive movement of the jaws and opisthotonos. The breathing seemed to be completely under the control of the instrument, and varied in fullness and frequency in proportion to the strength of the application. At one time, by way of experiment, the respiration was carried as high as thirty in a minute; the face becoming flushed and the perspiration streaming from the surface, but without any sign of consciousness. When the use of the battery was suspended, the breathing grew gradually less and less full and frequent, the extremities cool, and the system seemed again approaching a state of collapse, from which it was easily aroused by a renewed application of the battery. At one time the breathing fell in this way as low as four in a minute. Signs of consciousness first appeared at 10 o'clock, P. M., fourteen hours and a half after the opiate was taken. At that time she pushed away a cup of tea that was put to her mouth, and spoke incoherently. The electricity was applied at intervals during the night, and at 7, A. M., of the next day, consciousness was complete. In this case there seemed to be no special advantage in applying the electric current directly in the course of the nerves of respiration; the effect was more marked when the poles were placed in the hands of the patient. It seemed to act as a direct stimulant to the muscles of respiration. The instrument employed was a large Davis and Kidder's machine.

The President mentioned a case of paralysis, and diminution of the power of the special senses, coming on after diphtheria.—*Boston Med. and Surg. Journal.*

